# Pathogenic Genome Signatures That Damage Motor Neurons in Amyotrophic Lateral Sclerosis

**DOI:** 10.3390/cells9122687

**Published:** 2020-12-15

**Authors:** Ali Yousefian-Jazi, YunHee Seol, Jieun Kim, Hannah L. Ryu, Junghee Lee, Hoon Ryu

**Affiliations:** 1 Center for Neuroscience, Brain Science Institute, Korea Institute of Science and Technology, Seoul 02792, Korea; yousefian@kist.re.kr (A.Y.-J.); yseol@kist.re.kr (Y.S.); jieunkim@kist.re.kr (J.K.); 2 Department of Neurology, Boston University Alzheimer’s Disease Center, Boston University School of Medicine, Boston, MA 02118, USA; hr1@wellesley.edu; 3 VA Boston Healthcare System, Boston, MA 02130, USA

**Keywords:** amyotrophic lateral sclerosis, motor neuron, genome signature, cell damage, neurodegeneration

## Abstract

Amyotrophic lateral sclerosis (ALS) is the most frequent motor neuron disease and a neurodegenerative disorder, affecting the upper and/or lower motor neurons. Notably, it invariably leads to death within a few years of onset. Although most ALS cases are sporadic, familial amyotrophic lateral sclerosis (fALS) forms 10% of the cases. In 1993, the first causative gene (*SOD1*) of fALS was identified. With rapid advances in genetics, over fifty potentially causative or disease-modifying genes have been found in ALS so far. Accordingly, routine diagnostic tests should encompass the oldest and most frequently mutated ALS genes as well as several new important genetic variants in ALS. Herein, we discuss current literatures on the four newly identified ALS-associated genes (*CYLD*, *S1R*, *GLT8D1*, and *KIF5A*) and the previously well-known ALS genes including *SOD1*, *TARDBP*, *FUS*, and *C9orf72*. Moreover, we review the pathogenic implications and disease mechanisms of these genes. Elucidation of the cellular and molecular functions of the mutated genes will bring substantial insights for the development of therapeutic approaches to treat ALS.

## 1. Introduction

Amyotrophic lateral sclerosis (ALS) is a progressive neurodegenerative disease characterized by both upper and lower motor neuron degeneration, paralysis, and ultimately limiting survival from two to five years after onset [1]. Disease onset typically occurs in late middle-life with the mean age being 65 years. It results in relentless progressive muscle atrophy and weakness, ending with respiratory failure [2,3]. In addition, this neurodegenerative disorder has an estimated worldwide mortality rate of 30,000 patients per year [4]. ALS cases are estimated to occur in 2–3 per 100,000 individuals in Europe, and less than one in Asia [5], thus categorizing it as a rare disease. Furthermore, up to 10% of ALS-affected individuals have an affected family member or members with familial ALS (fALS), in which most have inherited the disease in an autosomal dominant manner [6]. The remaining ALS patients, with no clear genetic linkage, are called sporadic ALS (sALS) [7]. At present, mutations in over 50 genes have been shown to contribute to the ALS pathogenesis [8,9]. Some of them such as *SOD1*, *C9orf72*, *FUS*, and *TARDBP* were shown to present deleterious mutations, while other variants mostly found by association studies rarely occur in the less frequent genes [8,9,10]. Several studies have identified oxidative stress, glutamate excitotoxicity, apoptosis, neurofilament dysfunction, protein misfolding and aggregation, impairment of RNA processing, disrupted axonal transport, endosomal trafficking dysfunction, inflammation, and mitochondrial impairment as the molecular pathways which lead to the disease and indicate ALS pathogenesis. RNA metabolism and protein metabolism have been indicated as commonly proposed ALS pathogenic mechanisms [11].

Since the end of the 19th century, there have been many studies on ALS, but its pathogenesis is still unclear [4]. Many genetic variants that contribute to the missing heritability of ALS are yet to be discovered. Nowadays, the number of studies on finding novel ALS genes and their functions on disease pathogenesis is growing quickly [12,13,14,15,16,17,18,19,20]. This review discusses the current genetic understanding and landscape of ALS and summarizes its disease mechanisms. Here, we specifically review the genes involved in ALS in three categories, the most prevalent, the less frequent (≤1%), and the most recent ALS genes, and depict how genomic signatures turn into pathogenic molecules in ALS.

## 2. Genetics of ALS

**Figure 1:**
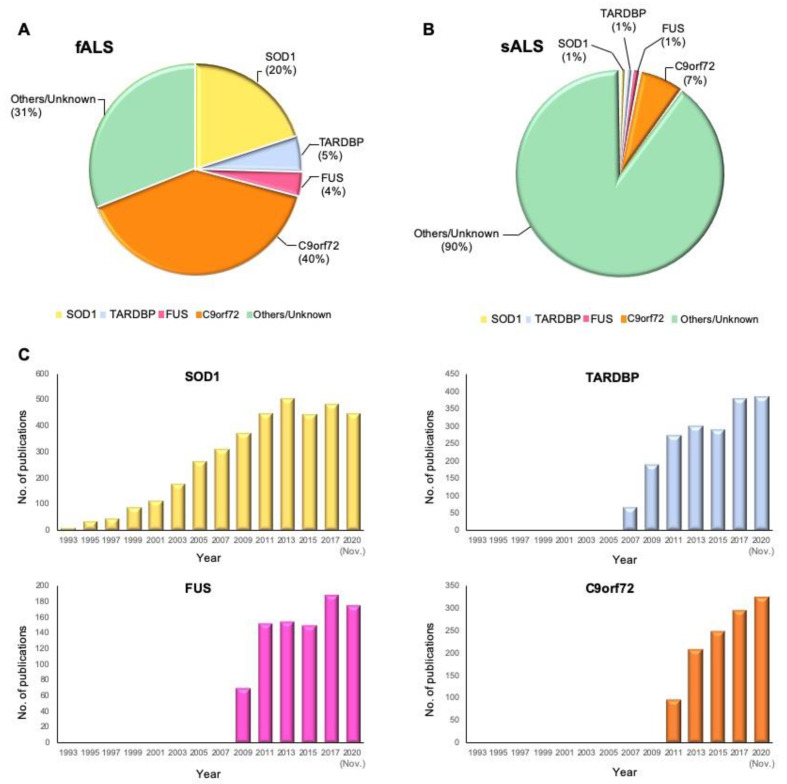
Proportional contribution of mutated genes in ALS and timeline of ALS gene study. (**A**) The proportion of mutant genes in fALS cases. (**B**) The proportion of mutant genes in sALS cases. (**C**) Timeline of ALS gene discoveries and researches for *SOD1*, *TARDBP*, *FUS*, and *C9orf72*. The *Y*-axis shows the number of publications in PubMed that include the terms “ALS” and “the gene name” by year until November 2020.

To date, 5–10% of all ALS cases suffer the known mutant genes [21,22]. *SOD1* is the most frequently mutant gene with 20% prevalence; mutant *TARDBP* and *FUS* in 5% and 4% of fALS cases, respectively, have been identified as well. In the United States and Europe, 40% of fALS cases suffer *C9orf72* variants, and the rest are caused by variants in other known or unknown genes (Figure 1) [23]. Simultaneously, variants in *C9orf72* and *SOD1* genes, which occur most frequently, constitute 7% and 1% of sALS cases, respectively [24]. To provide historical context, we illustrate the yearly number of publications from PubMed that include the terms “ALS” and “the gene name” in the title and abstract (Figure 1C). According to the timeline of gene discoveries, *SOD1*, *TARDBP*, *FUS*, and *C9orf72* are the first to fourth genes found with more than 1% occurrences in the patients in the years 1993, 2006, 2009, and 2011, respectively. Furthermore, there are some genes involved in ALS with less frequent variants (≤1%) (Table 1) and several recent genes over the past two years with relatively rare variants such as *CYLD*, *S1R*, *GLT8D1*, and *KIF5A*, which are precisely discussed in this review.

### 2.1. The Newly Identified ALS-Associated Genes (2018–2020)

Several novel ALS-associated genes have been proposed by the researchers over the past two years. Farhan et al. identified *DNAJC7* as a novel gene for ALS that encodes a member of the heat-shock protein family, HSP70, and has a key role in protein function such as protein folding and stabilization. Alteration of *HSP70* and *DNAJC7* gene expressions causes protein aggregation in ALS model [17]. Another gene, *WDR7*, was proposed by Course et al., in which the human-specific 69 bp variable number tandem repeat in the last intron of this gene may be associated with ALS. *WDR7* repeat expansions may act similar to the specific range of CAG repeat expansion numbers at *ATXN2,* which are enriched in ALS cases. It was shown that *WDR7* repeat expansion could form microRNAs, RNA aggregates, and lead to RNA editing [18]. Further study has shown that *ATXN1* overexpression disturbs TDP-43 nucleocytoplasmic transport, which leads to a decrease in the nucleocytoplasmic ratio of TDP-43. Hence, mislocalization and aggregation of TDP-43 can be considered the hallmark of ALS [19]. Finally, *ACSL5*, a neurotoxic A1 astrocyte-related gene, is upregulated in ALS cases. ACSL5 can induce A1 astrocytes, leading to motor neuron death and ALS progression. Overexpression of *ACSL5*, similar to the previously discovered gene *GPX3*, is associated with rapid weight loss in humans [8,20]. Although the mentioned genes above have been identified as ALS associated genes, the study on their contribution to ALS pathogenesis is still limited.

**Figure 2:**
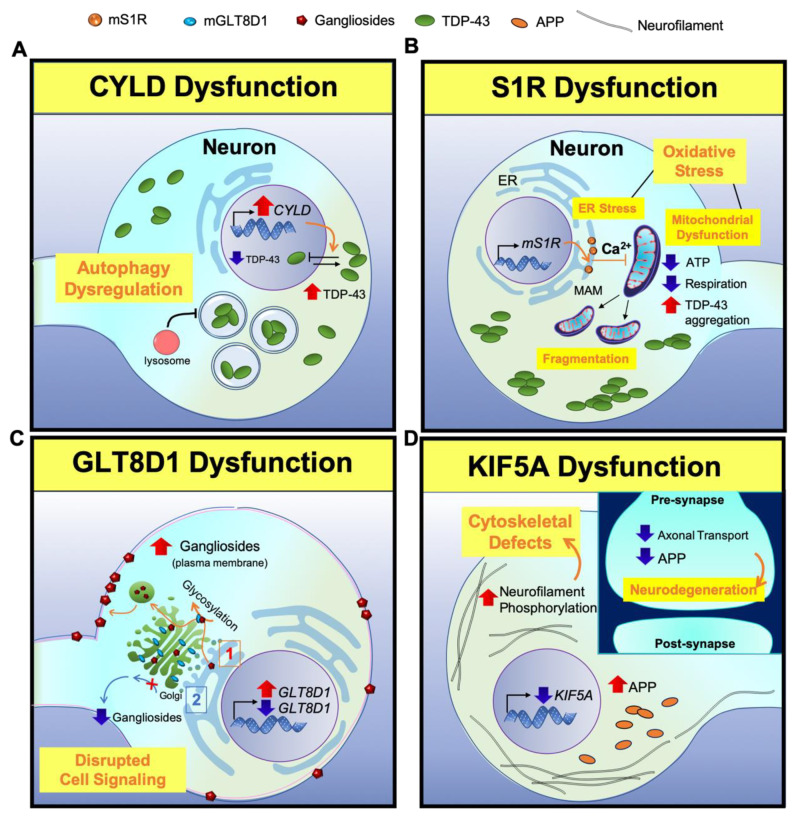
A scheme for illustrating neuropathogenesis of *CYLD*, *S1R*, *GLT8D1,* and *KIF5A* genes in ALS. (**A**) *CYLD*: Overexpression of CYLD inhibits the nuclear transport of TDP-43 from the cytoplasm, leading to the mislocalization of TDP-43. Lower TDP-43 level in the nucleus decreases the expression of ATG7, which is responsible for the fusion of lysosomes with autophagosomes. This results in a loss of autolysosome formation, causing a loss of autophagy function. (**B**) *S1R*: Mutant S1R leads to ER calcium and ATP depletion and perturbs mitochondrial dynamics and respiration. Subsequently, depletion of ATP leads to TDP-43-induced toxicity. (**C**) *GLT8D1*: (1) Mutant GLT8D1 increases the ganglioside signaling, leading to the transit of mature gangliosides to the cell membrane where they disrupt cell signaling. (2) On the other hand, knock down of *GLT8D1* impairs its glycosyltransferase activity from the Golgi and diminishes ganglioside signaling. (**D**) *KIF5A*: Lack of KIF5A expression disrupts axonal transport, accumulates phosphorylated neurofilaments and APP, and causes cytoskeletal defects in neurons. (Inlet) In contrast, the disruption of axonal transport reduces APP level in the synapse and triggers neurodegeneration.

Dobson-Stone et al. identified a novel missense variant in *CYLD* gene as the genetic cause of ALS in a large European Australian family [16]. Previously, they found a disease locus on chromosome 16 by genome-wide linkage analysis [56]. The missense variant in *CYLD* leads to alteration of CYLD immunoreactivity in the brain tissue [16], and shows two ALS-associated pathological phenotypes: an elevation of cytoplasmic TDP-43 level [57] and an impairment of autophagy function [58]. Overexpression of *CYLD* inhibited transport of TDP-43 into the nucleus from the cytoplasm. In the nucleus, TDP-43 controls the expression of ATG7, which mediates the fusion of lysosomes with autophagosomes. Decreased expression of ATG7 resulted in a loss of autolysosome formation [57,58,59]. Notably, overexpression of *CYLD* leads to the failure of autophagosome–lysosome fusion, causing the malfunction of autophagy [16,60] (Figure 2A).

*Sigma-1 receptor* (*S1R*), another potential therapeutic target gene in ALS discovered by Couly et al., regulates mitochondrial respiration and controls cellular defense against endoplasmic reticulum and oxidative stress [15]. S1R is mainly located in a special compartment of the Endoplasmic reticulum (ER) called the mitochondria-associated membrane (MAM) and has a role in ATP production [61]. S1R also protects TDP-43-induced toxicity by rescuing ATP production. ATP binds to the N-terminal domain of TDP-43 to enhance its oligomerization, and prevents the aggregation of TDP-43 into its toxic form [62]. Mutant *S1R* (m*S1R*) leads to ATP depletion and perturbs mitochondrial dynamics and respiration [15]. Therefore, it is demonstrated that an overexpression of m*S1R* is neurotoxic, leading to mitochondrial dysfunction, thus highlighting the role of *S1R* in ALS therapy [15] (Figure 2B). A recent study demonstrated that m*S1R* leads to Drosophila photoreceptor organization alteration and spontaneous walking behavior [15]. Moreover, the motor performance in S1R knockout mice diminished including muscle weakness, axonal degeneration, and loss of motor neuron [63]. Finally, the protective role of S1R has been dementated in the ALS mice (G93A) model with S1R knockout by behavioral and longevity experiments [64].

Cooper-Knock et al. proposed variants in the *GLT8D1* gene which are recognized to be causal in ALS [14,65]. GLT8D1, expressed within the Golgi, is a member of the glycosyltransferase family 8 involved in catalyzing the transfer of glycosyl groups. In addition, gangliosides are synthesized in the ER, which are remodeled to maturation from the cis-Golgi to the trans-Golgi network via glycosylation by GLT8D1 [66]. The mature gangliosides, which are moved to the cell membrane, are involved in cell signaling [67] and produce neuroinflammation in motor neurons. ALS-associated mutations in *GLT8D1* prevent the normal activity of glycosyltransferase enzyme and negatively impacts ganglioside signaling. The overexpression of mutant *GLT8D1* increases ganglioside signaling, which leads to the transit of mature gangliosides to the cell membrane where they disrupt cell signaling. In contrast, the knock down of *GLT8D1* impairs its glycosyltransferase activity from the Golgi and diminishes ganglioside signaling. The previous study demonstrated that both knock down and overexpression of mutant *GLT8D1* induce motor neuron dysfunction and produce cytotoxicity in zebrafish consistent with ALS. In the previous studies, genetic variants in this gene showed a significant increase in disease severity and cytotoxicity in ALS patients [14,68] (Figure 2C).

Thus far, several studies have described the clinical evolution and the genetic findings of the *KIF5A* gene in sALS and fALS [12,13,69,70]. The genome-wide association study comparing 20,806 ALS cases and 59,804 controls discovered *KIF5A* as a novel gene associated with ALS [13]. Independently, rare variant analysis was conducted on 426 patients with fALS and 6137 control subjects, which identified enriched *KIF5A* splice-site variants in the cases. The genetic variants associated with ALS are located at the C-terminal cargo-binding tail domain of the *KIF5A* gene, which is also expressed in neurons. Considering the contribution of axonal transport deficits in the pathogenesis of motor neuron degeneration [71,72,73], variants in *KIF5A* disrupt axonal transport and amyloid precursor protein (APP) depletion in the synapse, which causes neurodegeneration. Therefore, a lack of KIF5A expression, which transports cargo by binding to distinct adaptor proteins, has been associated with the accumulation of phosphorylated neurofilaments and APP in the neuronal cell, which leads to cytoskeletal defects [13,74]. Moreover, previous studies confirmed the involvement of intracellular transport processes and strengthened the role of cytoskeletal defects of mutated *KIF5A* in ALS pathogenesis (Figure 2D). The first genotype–phenotype relationship showed that ALS patients with KIF5A loss-of-function mutations correlated with disease onset at an earlier age and longer survival [13]. The second was proposed by Brenner et al., in which adult onset, rapid progression, and early death were shown in the patients with KIF5A splice-site mutations [12].

### 2.2. The Old Genes with Important Contribution to ALS

**Figure 3:**
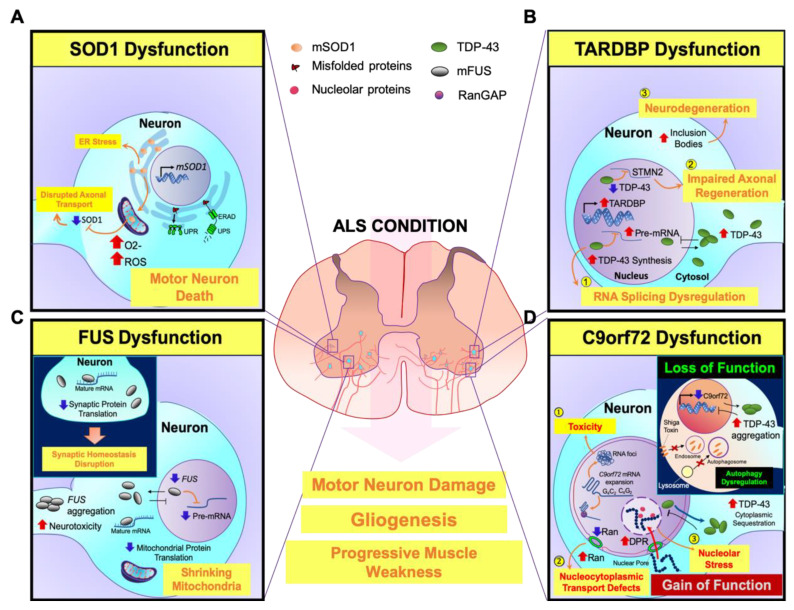
A scheme for illustrating neuropathogenesis of mutant genes (*SOD1*, *TARDBP*, *FUS*, and *C9orf72*) with major contribution to ALS. (**A**) *SOD1*: Mutant SOD1 (mSOD1) leads to oxidative stress by elevating free radicals that damage protein folding and impair axonal transport in motor neurons. Additionally, misfolded mSOD1 protein is not able to transport across mitochondrial membranes and accumulates onto the outer mitochondrial membrane, triggering motor neuronal death. On the other hand, mSOD1-induced ER stress results in the activation of UPR and ERAD, causing motor neuronal damage. (**B**) *TARDBP*: (1) Mutant TDP-43 (mTDP-43) is aggregated in the cytosol and TDP-43 dysfunction abnormally accumulates mRNA in the nucleus. (2) The loss of nuclear TDP-43 function leads to inhibition of TDP-43 binding to *STMN2* RNA and mis-splicing of *STMN2* RNA, which subsequently inhibits axonal regeneration of motor neurons. (3) On the other hand, upregulation of cytoplasmic TDP-43 forms inclusion bodies and it further propagates to neighbor cells, causing motor neuronal degeneration. (**C**) *FUS*: Mutant FUS (mFUS) disrupts nucleocytoplasmic transport and leads to its depletion from the nucleus. Then, mFUS aggregates in the cytoplasm and induces neurotoxicity. mFUS may also disrupt translation of transcripts associated with mitochondrial function, which leads to mitochondrial shrinkage. (Inlet) Moreover, mFUS binds to mature mRNAs in the cytoplasm and affects mRNA translation. Suppressed protein translation impairs the dendrites and axon terminals. Inhibition of local intra-axonal protein translation, that are required for synaptic maintenance and function, causes synaptic defects and dysfunctions. (**D**) *C9orf72*: (1) The nuclear RNA foci are generated by the aggregation of repeat-containing *C9orf72* RNAs in the nucleus and cause neurotoxicity. (2) Sequestration of RanGAP by G4C2 RNA disrupts the nucleocytoplasmic transport function. The loss of nuclear Ran depletes nuclear TDP-43 levels and elevates cytoplasmic TDP-43 levels. (3) Furthermore, the imported dipeptide repeats (DPRs) into the nucleus are associated with nucleolar proteins and cause nucleolar stress. (Inlet) The loss of C9orf72 function in endosomal trafficking regulation by interactions with nuclear pore complex proteins eventually increases cytoplasmic TDP-43 inclusions. Furthermore, reduction of *C9orf72* expression inhibits Shiga toxin transportation from the plasma membrane to the Golgi apparatus, and alters the ratio of LC3, an autophagosome marker, leading to autophagy dysregulation.

*SOD1*, *TARDBP*, *FUS*, and *C9orf72*, as the oldest and most frequently mutant ALS genes, are described and their function in ALS pathogenesis are illustrated (Figure 3).

#### 2.2.1. SOD1

**Figure 4:**
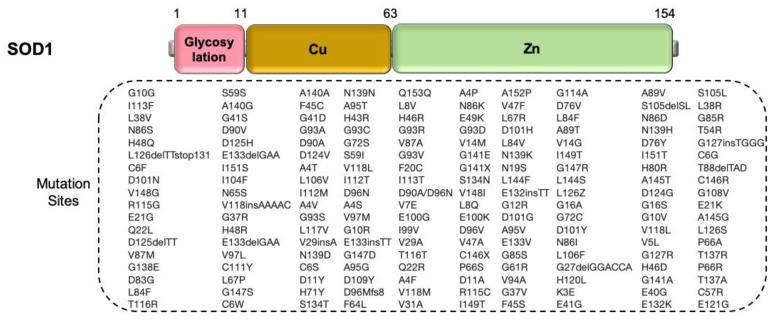
A scheme for exhibiting variant sites in SOD1 protein. (Top) the SOD1 protein structure; (Bottom) 180 SOD1 variants shown in ALS cases. Data were taken from the ALS online database (ALSoD) [79]. The variations are only identified on the amino acid sequence of the protein, and not all are proven to be pathogenic for ALS.

The *SOD1* gene (encoding superoxide dismutase 1 (Cu/Zn)), which maps to chromosome 21q22.1, was the first gene identified in fALS [75]. According to a recent genome-wide meta-analysis, approximately 15–30% of fALS and less than 2% of sALS cases have been identified to have the pathogenic variants of SOD1 [24]. Currently, 180 genetic variants have been discovered to affect the functional domains of the *SOD1* gene including D90A, which is identified as the most frequent missense variant (Figure 4). Recently, the SOD1 homozygous truncating variant, c.335dupG, with total absence of SOD1 activity was identified in ALS affected patients [76]. Depending on the genetic variant, different molecular morphological changes can result, and patients with *SOD1*-related ALS who harbor particular variants have distinct clinical features [10,77]. For example, patients with the A4V, H43R, L84V, G85R, N86S, and G93A variants show rapid disease progression and shorter survival times, while patients carrying the G93C, D90A, or H46R variants show a longer life expectancy [78].

SOD1 is an antioxidant homodimeric protein of 153 amino acids, containing one copper and one zinc atom [22,80]. It can be localized from nucleus to cytosol or mitochondrial intermembrane space. The function of SOD1 is to protect cells from reactive oxygen species toxicity. Both copper and zinc play specific roles in SOD1 activity and structural stability, respectively, and are directly involved in the deactivation of toxic superoxide radicals [22,81,82]. Previous studies have supported that SOD1-ALS is caused by a gain of function, increasing the function of producing free radicals [83]. Mutant SOD1 modifies the oxidative activity, which causes accumulation of toxic hydroxyl radicals [77,84]. Accumulation of free radicals in the intermembrane space of the mitochondria leads to mitochondrial damage and disrupted protein folding, significantly affecting distal axons of motor neurons [82,85,86]. ER is a cellular compartment including chaperone-assisted proteins to help fold proteins. Mutant SOD1 activates ER stress which leads to activation of the unfolded protein response (UPR) and ER-associated degradation (ERAD). Resulting in the refolding of misfolded proteins and export of misfolded proteins from the ER to the ubiquitin proteasome system (UPS) for degradation, respectively. Long activation of the ER can cause pro-apoptotic consequences [87]. On the other hand, mutations in SOD1 impairs axonal transport. Therefore, misfolded SOD1 is not able to transport across the mitochondrial membranes and accumulates in the outer mitochondrial membrane, triggering the mitochondrial-dependent cell apoptosis program [88] (Figure 3A).

Pansarasa et al. identified a difference between protein expression and mRNA levels in wild-type SOD1 and sALS patients, and then proved their hypothesis on translocation and re-localization of the missing SOD1 in the nucleus. Furthermore, they found that the higher amounts of soluble SOD1 in the nucleus are positively correlated with a longer duration of disease, indicating a possible protective role of SOD1 [89]. Therefore, SOD1 can be considered as a good target for ALS therapy. In this perspective, riluzole, a sodium channel blocker and glutamate release inhibitor, has been applied to improve ALS symptoms and is approved by the FDA for treating ALS [90]. shRNA, miRNA, and RNAi have been evaluated for mediating SOD1 silencing in transgenic mice, which are under investigation for ALS treatment [91]. Moreover, SOD1-ALS patients show some features and clinical characteristics that are slightly different compared to other ALS patients such as earlier age of onset, longer duration of disease, and motor symptoms that begin more often in the lower limbs [92,93].

#### 2.2.2. TARDBP

**Figure 5:**
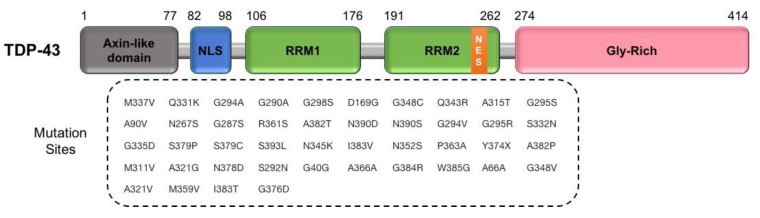
A scheme for exhibiting variants sites in TDP-43 protein: (Top) the TDP-43 domain structure; and (Bottom) the 44 TDP-43 variants shown in patients with ALS. NLS, nuclear localization signal; NES, nuclear export signal; RRM, RNA recognition motif.

In the 1990s, Leigh et al. concluded that neuronal cytoplasmic ubiquitinated inclusions were found in the spinal cord samples from ALS patients [94]. In 2006, TAR DNA-binding protein 43 (TDP-43) was discovered as the main reason for the protein aggregation in sALS cases [57,95]. Later, in 2008, several studies identified genetic variants in the *TARDBP* gene and the deviation of TDP-43 as a primary cause of ALS and neurodegeneration [26,96,97,98,99]. To date, over 40 variants have been identified in various ethnic groups with around 5% in fALS and up to 2% in sALS cases [100]. The majority of variants are missense variants located in the glycine-rich region at the transcript carboxy-terminal, which interacts with other heterogeneous ribonucleoproteins (Figure 5). The carboxy-terminal region is also involved in pre-mRNA splicing regulation [57,101]. Further studies on both fALS and sALS patients have shown the existence of TDP-43 in cytoplasmic aggregates of those without pathogenic variants in the *TARDBP* gene and carrying *C9orf72* hexanucleotide repeat expansions [102,103,104].

TARDBP/TDP-43 is a DNA/RNA binding protein composed of 414 amino acids that are encoded by *TARDBP/TDP43* gene. TDP-43 belongs to the ribonucleoprotein family and has various functions such as gene transcription, microRNA processing, RNA splicing and stabilization, and mRNA transport [105]. TDP-43 has both nuclear localization and export signals and continuously shuttles between the nucleus and cytoplasm [106,107]. Any interference in the normal intracellular trafficking of TDP-43 between the cytoplasm and nucleus can result in cytoplasmic aggregation and the loss of nuclear TDP-43 function in regulating transcription, splicing, and mRNA stability [108,109,110]. The challenging question about the role of TDP-43 in ALS is whether a toxic gain of function of cytoplasmic aggregates or a loss of its normal function in the nucleus is responsible for disease. During cellular stress, TDP-43 plays a significant role in controlling mRNA stability, translation, and nucleocytoplasmic transport [111]. In the case of ALS, while the loss of nuclear TDP-43 function leads to dysmorphic nuclear shape, deregulation of the cell cycle, and apoptosis [112], an overexpression of TDP-43 leads to abnormal mRNA accumulation in the nucleus, cytoplasmic accumulation [113,114], and a lost normal functioning in the nucleus. Furthermore, the upregulation of cytoplasmic TDP-43 forms the inclusion bodies and the capacity to propagate among cells as a “prion-like” protein, a significant reason for the neurodegeneration observed in motor neurons [115]. However, several studies used cultured cells, animal models, and patients autopsies, demonstrating how cytoplasmic TDP-43 aggregates have an important role in motor neuronal death and neurodegeneration observed in ALS patients [115,116]. Melamed et al. proposed that ALS is associated with loss of nuclear TDP-43 [117]. Their results confirm that reduction of nuclear TDP-43 inhibits regeneration of motor axons, which is the consequence of reduction in stathmin-2 (STMN2), an essential protein for axonal growth and maintenance (Figure 3B) [117,118].

The discovery of TARDBP/TDP-43 and FUS, two RNA-binding proteins, highlights the importance of RNA processing in ALS pathogenesis. Previous studies have focused on understanding how exactly these mutant proteins disrupt RNA transcription and modification [119]. As expected, upregulation of TARDBP/TDP-43 in motor neurons increased ALS risk by altering RNA splicing and stability [120,121,122]. On the other hand, lack of TARDBP/TDP-43 in the forebrains of mice resulted in age-dependent brain atrophy by downregulating protein Tbc1d1 in skeletal muscles, leading to compromised neuronal function [121,122]. In addition, Tsao et al. developed a *Tardbp* knockout mice model and showed a decrease in TARDBP/TDP-43 levels as well as a loss in body weight caused by an increase in fat oxidation and acceleration of fat loss in adipocytes [121]. Another study in *Tardbp* knock-in ALS mice indicated that mutant TDP-43 causes early-stage and dose-dependent motor neuron degeneration [123]. A recent study demonstrated that ALS patient-derived TARDBP/TDP-43 mutation at the carboxyl-terminal domain (M337V) causes splicing deregulation without motor neuronal degeneration in mice [124].

#### 2.2.3. FUS

**Figure 6:**
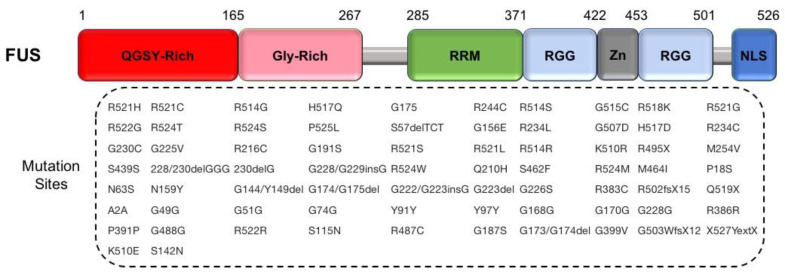
A scheme for exhibiting variants sites in FUS/TLS protein: (Top) the FUS domain structure; and (Bottom) the 72 FUS variants shown in patients with ALS. RGG, Arg-Gly-Gly.

The next discovered gene that may rival the impact of TDP-43 on ALS research is the *FUS* gene (also known as TLS), which maps to the ALS6 locus on chromosome 16p11.2. Variants in the *FUS* gene are known to be causal for fALS [41,125]. The disease onset of ALS patients with ALS6 variants spans across a wide range of ages (from 26 to 80 years old) with a mean duration of around 33 months [80]. Over 70 variants in *FUS* have been identified in ALS patients, some of which have proved to be causal (Figure 6). ALS patients with *FUS* variants have a shorter life span, although extensive intrafamilial variability has been observed [126]. Waibel et al. showed two truncating *FUS* variants associated with consistent early onset and an aggressive disease course [127]. *FUS* variants have been reported to contribute 4% and 1% in fALS and sALS cases overall, respectively [128]. The most recent *FUS* variants were indicated as the most frequent cause of early-onset ALS (at ages less than 35 years) in German fALS patients with 8.7% frequency [127,129].

FUS is an RNA binding protein encoded by a ubiquitously expressed gene and composed of 526 amino acids. FUS, EWSR1, and TAF15 belong to FET families, which are involved in transcription and alternative splicing by interacting with the transcription pre-initiation complex and various splicing factors. Under normal physiological conditions, FUS is mostly localized in the nucleus, but it shuttles to the cytoplasm and functions in nucleocytoplasmic transport [130]. Therefore, mutant FUS (mFUS) disrupts nucleocytoplasmic transport, leads to a depletion in the nucleus, aggregates in the cytoplasm, and causes neurotoxicity [131]. Mutant FUS binds to mature mRNAs in the cytoplasm, unlike WT FUS, which binds to precursor mRNAs. Abnormal binding of mFUS affects mRNA expression but has only modest expression changes. Mutant FUS not only suppresses global protein translation but also local protein translation, impairing the dendrites and axon terminals. Considering FUS as a part of RNA transport granules and its role in activated synapses [132,133], there would be defects in synaptic homeostasis and dysfunction in cells suffering from FUS mutations [134,135]. Diminishing of the proteins required for synaptic maintenance and function may lead to an ALS phenotype [136]. Furthermore, the gene binding profile has been altered in mFUS, which leads to neurotoxicity and mitochondrial size reduction due to disruption of the translation of transcripts associated with mitochondrial function [137] (Figure 3C).

In a study developed in 2015, while homozygous FUS knockout mice survived into adulthood, they had the phenotypes related to neuropsychiatric and neurodegenerative conditions, but different from ALS [138]. In further study, a model with conditionally removed FUS developed from the motor neurons of mice showed no significant effect on motor neuron survival or function. This suggests that a loss of FUS is not a sufficient cause for ALS [139]. However, Sasayama et al. found another layer of results when they used Drosophila FUS knockdown models [140]. They showed that decreasing the expression of Drosophila ortholog of FUS plays an important role in the degeneration of motoneurons and locomotive disability in the absence of abnormal cytoplasm aggregates. This suggests that the pathogenic mechanism of FUS-ALS can be considered as a loss of physiological FUS function in the nucleus rather than cytoplasmic FUS aggregate toxicity [140]. Moreover, mFUS transgenic rats developed progressive paralysis due to a loss of neurons in the cortex and hippocampus [141]. Later, Chen et al. showed that age-dependent progressive motor neurons were damaged when WT, R524S, or P525L mFUS were over-expressed in photoreceptors [142]. Recently, it was demonstrated that mFUS causes accumulation of NEAT1 isoforms and paraspeckles, which contribute to degenerating spinal motor neurons [143].

#### 2.2.4. Chromosome 9 Open Reading Frame 72 (C9orf72)

**Figure 7:**
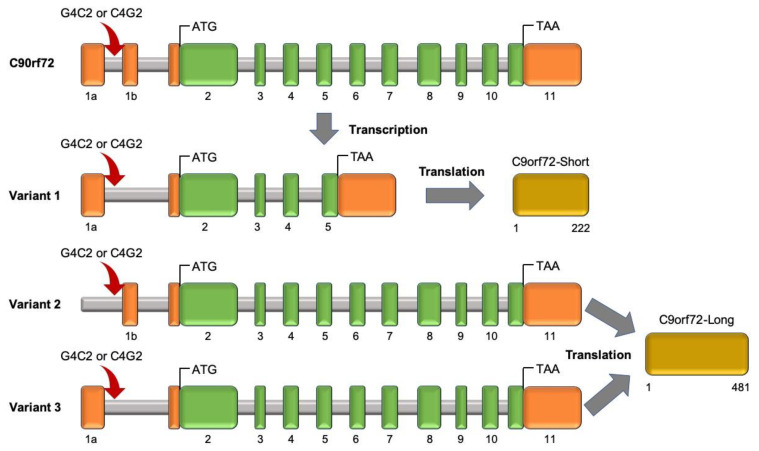
*C9orf72* gene structure, transcript variants, and protein isoforms. The *C9orf72* gene consists of 11 coding exons (green) and non-coding exons (orange). The G4C2 or C4G2 HRE is located in the first intron of variants 1 and 3 and within the promoter region of variant 2. This figure was adapted from Balendra and Isaacs’s study [146].

Hexanucleotide repeat expansion (HRE), GGGGCC (G_4_C_2_), in the non-coding region of the *C9orf72* gene was found as the most common inherited cause of ALS in European cohort in 2011 [45,144]. Renton et al. proposed that the G_4_C_2_ HRE in the first intron on the affected haplotype in ALS patients is larger compared to healthy subjects (less than 30 HREs) [144,145] (Figure 7). However, increasing the repeat expansions is thought to be pathogenic. The exact cut-off between normal alleles and pathogenic expanded alleles is still unclear. In European cohorts, this HRE frequently occurs approximately in 40% and 7% of fALS and sALS, respectively, but is less frequent in Asian cohorts [24].

The known mechanisms for how HREs cause the disease can be categorized into two primary mechanisms [147]. First, mechanisms driving C9orf72 gain of function where RNAs containing G4C2 and C4G2 expanded repeats are bi-directionally transcribed and then aggregated in the cell nucleus. Dipeptide repeat proteins (DPRs) can be generated from repeat-containing RNAs that leave the nucleus. The imported DPRs into the nucleus can bind to the nucleolar proteins and cause nuclear stress [147,148,149,150]. On the other hand, RNA repeat-expansions can be retained in the nucleus and generate RNA foci, which has an important role in RNA-binding proteins, changes in RNA processing, nucleocytoplasmic transport impairment, and toxicity in the cell. C9orf72 RNA can sequester nucleolar proteins, bind to nuclear pore complex proteins, and disrupt nucleocytoplasmic transport. In addition, sequestration of RanGAP by the G4C2 RNA causes a reduction in the nuclear–cytoplasmic (N/C) distribution of Ran GTPase (Ran) and disrupts functional nucleocytoplasmic transport [131,151]. The nucleocytoplasmic trafficking correlated with mislocalization of TDP-43 in the cytoplasm. The second is C9orf72 loss-of-function mechanisms. In this mechanism, HRE leads to disrupted transcription, downregulation of *C9orf72*, and a loss of function. Previous studies showed the involvement of C9ORF72 in endosomal trafficking regulation as well as the inverse correlation between C9-isoform interactions with the nuclear pore complex and TDP-43 cytoplasmic inclusion levels. Then, a strong reduction of C9orf72 expression caused an inhibition of Shiga toxin transportation from the plasma membrane to the Golgi apparatus and altered expression of the autophagosome marker LC3 ratio [152,153]. Therefore, neurons from ALS patients with variants in C9orf72 have increased sensitivity to autophagy inhibition, suggesting that reductions in gene levels can lead to cellular distress [146] (Figure 3D).

In C9orf72-ALS patients, the age of onset is mostly between 30 and 70 years and bulbar onset has been more frequently observed [154]. Not only ALS and FTD but also parkinsonism and psychotic symptoms can be caused by C9orf72 expansions [155,156]. Considering the decreasing levels of *C9orf72* mRNA and proteins in ALS patients with repeat expansions [157,158], Koppers et al. tested the validity of this hypothesis by developing a C9orf72 conditional knockout mouse model. However, evidence of motor neuron degeneration or motor deficits were unobserved [159]. A recent report case study highlights the phenotypic variability, including age of onset, within a family with the *C9orf72* repeat expansion [160].

### 2.3. The Less Frequently Mutated Genes in ALS

Nowadays, by developing next-generation sequencing technologies, rare genetic variants were discovered in several studies. However, the specific link between gene variants and disease mechanisms is sometimes unclear because of the small number of patients carrying those variants. Functions of the discovered genes can be categorized into four categories: (1) genes that influence RNA processing such as *ANG* [31], *SETX* [36], *MATR3* [47], *ATXN2* [30], *TAF15* [161], *EWSR1* [53,162], *ELP3* [51], *hnRNPa1*, *hnRNPA2B1* [29], *ATXN1* [163], and *GLE1* [164]; (2) genes involved in protein trafficking and degradation such as *ALS2* [42], *VAPB* [43], *CHMP2B* [46], *FIG4* [35], *UBQLN2* [55], *SQSTM1* [37], *SIGMAR1* [33], *OPTN* [21], *VCP* [165] and *TBK1* [166]; (3) genes that influence cytoskeletal and axonal dynamics such as *DCTN1* [167], *PFN1* [48], *SPG11* [27], *TUBA4A* [50], *NEFH* [168], *PRPH* [49], *NEK1* [169], *C21orf2* [170]; and (4) *CHCHD10* [171] and *C19orf12* [172] involved in mitochondrial dysfunction.

## 3. Conclusions

Although we have come a long way since proposing *SOD1* as the first ALS gene 27 years ago, the causative pathogenic mechanisms in ALS remain obscure. The function of mutant *CYLD*, *S1R*, *GLT8D1*, and *KIF5A* as the recently discovered genes involved in ALS pathogenesis is illustrated in Figure 2. In addition, Figure 3 provides previous findings of the genetic effects of mutant *SOD1*, *TARDBP*, *FUS*, and *C9orf72* and the fundamental mechanisms of ALS pathogenesis. According to the genes and genetic functions reviewed in this paper, multiple factors are involved in ALS disease development and progression, in which the most commonly proposed ALS pathogenic mechanisms are RNA metabolism and protein metabolism. Furthermore, genetic and phenotypic variants between patients make it difficult to draw general conclusions on ALS pathogenesis and predict the future outcomes in ALS research. However, further research on finding novel genes, gene modifiers, and their molecular pathways might improve our understanding about this neurodegenerative disorder, which is still fatal. In addition, new therapeutic strategies, by either the identification of shared disease pathways or targeted therapies known for genetic variants can be developed by proposing novel ALS causal genes. As of now, riluzole is the only successfully established therapy for ALS that exerts transient effects on cortical and axonal hyperexcitability [173], whereas several drug trials targeting glutamatergic neurotransmission have been unsuccessful [174]. Regarding the irreversibility of genetic variants, developing therapeutic approaches is difficult and specific drugs that target the treatment of ALS are quite limited [175]. Considering how genetic variants result in epigenetic modifications and how epigenetic alterations are reversibly modulated in various neurodegenerative disorders such as Alzheimer’s disease and Huntington’s disease, it would be reasonable to design therapeutic approaches that target the epigenetic components to ameliorate the onset and symptoms of ALS [176,177,178]. Therefore, in future studies, a combination of treatments that modulate the multiple targets of epigenetic components could be the more effective therapeutic strategy for treating ALS.

## Figures and Tables

**Table 1:** Genes known to carry ALS-causing mutations.

Gene	Cases/Control	Frequency (Familial)	Frequency (Sporadic)	Reference (s)
*SOD1*	2874/6405	20%	1%	[25]
*TARDBP*	526/1262	4%	1%	[26]
*OPTN*	673/781	<1%	<1%	[21]
*SPG11*	3056/900	<1%	<1%	[27]
*VCP*	288/1569	1%	1%	[28]
*hnRNPA1*	517/625	<1%	<1%	[29]
*ATXN2*	915/980	<1%	<1%	[30]
*ANG*	1629/1264	<1%	<1%	[31]
*CHCHD10*	4853/1991	<1%	<1%	[32]
*SIGMAR1*	158/1100	<1%	<1%	[33,34]
*FIG4*	473/558	<1%	<1%	[35]
*SETX*	49/100	<1%	<1%	[36]
*SQSTM1*	546/738	1%	<1%	[37]
*TBK1*	252/827	1%	<1%	[38]
*NEK1*	1022/7315	1%	1%	[39]
*TAF15*	357/1100	<1%	<1%	[40]
*FUS*	583/1446	4%	1%	[41]
*ALS2*	42/533	<1%	<1%	[42]
*VAPB*	24/400	<1%	<1%	[43]
*NEFH*	530/447	<1%	<1%	[44]
*C9orf72*	696/909	40%	7%	[45]
*CHMP2B*	400/640	<1%	<1%	[46]
*MATR3*	108/1051	<1%	<1%	[47]
*PFN1*	274/1089	<1%	<1%	[48]
*PRPH*	189/380	<1%	<1%	[49]
*TUBA4A*	363/5510	1%	<1%	[50]
*ELP3*	781/702	<1%	<1%	[51]
*DCTN1*	333/200	<1%	<1%	[52]
*EWSR1*	817/1082	<1%	<1%	[53]
*GLE1*	933/190	<1%	<1%	[54]
*UBQLN2*	200/928	<1%	<1%	[55]
